# Validity of intracerebral haemorrhage volume assessment: comparison of fully automated segmentation analysis with manual ABC/2 and semi-automated measurement

**DOI:** 10.1093/esj/aakaf020

**Published:** 2026-01-01

**Authors:** Nadia H C Colmer, Rahman Fakhry, Yvette de Haan, Bridget A Schoon, Ruben M van de Wijdeven, Charlotte C Kik, Sanne Steltenpool, Milad Raufi, Can Yesildal, Renske Gahrmann, Catharina J M Klijn, Diederik W J Dippel, Ruben Dammers

**Affiliations:** Department of Neurosurgery, Erasmus University Medical Center, Rotterdam, The Netherlands; Erasmus MC Stroke Center, Rotterdam, The Netherlands; Department of Neurosurgery, Erasmus University Medical Center, Rotterdam, The Netherlands; Department of Neurosurgery, Erasmus University Medical Center, Rotterdam, The Netherlands; Erasmus MC Stroke Center, Rotterdam, The Netherlands; Department of Neurology, Erasmus University Medical Center, Rotterdam, The Netherlands; Erasmus MC Stroke Center, Rotterdam, The Netherlands; Department of Neurology, Erasmus University Medical Center, Rotterdam, The Netherlands; Department of Neurosurgery, Erasmus University Medical Center, Rotterdam, The Netherlands; Department of Radiology and Nuclear Medicine, Erasmus University Medical Center, Rotterdam, The Netherlands; Department of Radiology, Elisabeth-TweeSteden Hospital, Tilburg, The Netherlands; Department of Neurosurgery, Erasmus University Medical Center, Rotterdam, The Netherlands; Department of Neurosurgery, Erasmus University Medical Center, Rotterdam, The Netherlands; Department of Radiology and Nuclear Medicine, Erasmus University Medical Center, Rotterdam, The Netherlands; Department of Neurology, Donders Institute for Brain, Cognition and Behaviour, Radboud University Medical Centre, Nijmegen, The Netherlands; Erasmus MC Stroke Center, Rotterdam, The Netherlands; Department of Neurology, Erasmus University Medical Center, Rotterdam, The Netherlands; Department of Neurosurgery, Erasmus University Medical Center, Rotterdam, The Netherlands; Erasmus MC Stroke Center, Rotterdam, The Netherlands; Expertise Center for Complex Micro(vascular) Surgery Rotterdam, Rotterdam, The Netherlands

**Keywords:** intracerebral haemorrhage, artificial intelligence, volume measurement

## Abstract

**Introduction:**

In patients with spontaneous Intracerebral haemorrhage (ICH), ICH volume is associated with neurological and functional outcome and it is an important factor in neurosurgical decision-making. This study aims to assess the agreement between automated ICH volume measurement, the ABC/2 method and semi-automatic segmentation.

**Patients and methods:**

We retrospectively collected data from 300 consecutive adult patients (November 2018–June 2023) with spontaneous, supratentorial ICH, with a symptom onset-to-CT time ≤ 24 hours. We measured ICH volumes with automated StrokeViewer software, the manual ABC/2 method and semi-automated Brainlab software (reference standard). We performed a Bland–Altman analysis to compare measurements, considering a deviation of 10% or less from the reference standard as clinically acceptable.

**Results:**

The median age was 69 years (IQR, 57–76), 124 (41.3%) were women and the median NIHSS was 18 (IQR, 11–23). Median ICH volume was 26.0 mL (IQR, 9.3–59.2) (Brainlab). StrokeViewer also segmented hyperdense structures other than ICH, but occasionally, it only segmented part of the ICH accurately. The mean absolute differences were 11.2 mL (limits of agreement [LoA] −34.3 to 56.7) between StrokeViewer and Brainlab, −0.42 mL (LoA −65.0 to 64.9) between StrokeViewer and ABC/2 and 10.1 mL (LoA −36.2 to 56.4) between ABC/2 and Brainlab.

**Conclusion:**

There is substantial disagreement between the 3 methods for the measurement of ICH volume. Considering a clinical limit of acceptance of 10% or less, neither StrokeViewer nor ABC/2 agreed with our reference standard. Therefore, StrokeViewer results should not be used for volume-based clinical decisions without visual confirmation of adequate segmentation.

## Graphical Abstract

**Graphical abstract:**
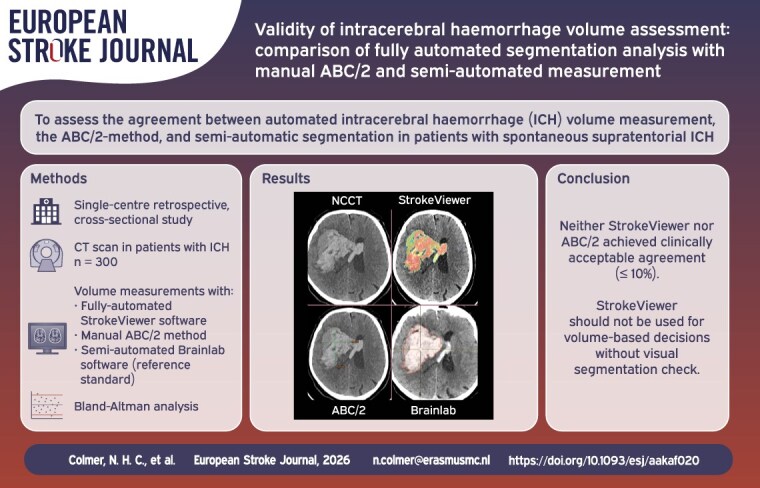

## Introduction

ICH constitutes approximately 28% of all strokes worldwide and is the stroke subtype with the highest mortality.^1^ The 30-day case fatality rate is approximately 35% and only about 31% of patients experience good functional outcome (mRS 0–2) after 3–12 months.^2^ ICH volume is an important prognostic factor for outcome, as larger haematoma volumes are associated with increased 30-day mortality and poor long-term neurological and functional outcome.^3^^,^^4^ Additionally, ICH volume is an important criterion when considering neurosurgical treatment.

There are various ways to estimate ICH volume. The most widely used method for haematoma volume measurement is ABC/2.^5^ This manual method is a relatively quick way to estimate ICH volume and is therefore frequently used in clinical practice and research settings. It requires the measurement of the diameter of the haematoma in 3 directions (A, B, C), perpendicular to each other, and calculation of haematoma volume with the formula (A·B·C)/2. The ABC/2 method is known to be less accurate for more irregular haematomas, since the equation is based on the assumption that the shape of the haematoma is a relatively regular ellipsoid.^6^

Another method for measuring haematoma volume is semi-automatic segmentation (eg, SmartBrush, Brainlab), which is commonly used for neurosurgical planning and navigation in daily practice. Although semi-automatic segmentation is more accurate than the ABC/2 method and widely considered as a reference standard, it is time-consuming and not always available in acute settings.^7^^,^^8^

Many new techniques for automatic detection have been developed recently.^9^ One of the commercially available packages is StrokeViewer, which was developed for analyses of imaging in ischaemic stroke. The application consists of AI stroke algorithms for ASPECTS evaluation on non-contrast-enhanced CT (NCCT) of the head, a deep learning convolutional neural network to detect large vessel occlusions (LVOs) in the anterior circulation and analysis of CT perfusion images. While the StrokeViewer app was developed for image analysis in ischaemic stroke, it can also provide detection and volume measurement of ICH. Rapid availability of these results following CT image analysis with the software allows for integration into acute-phase clinical decision-making.^10^^,^^11^ This potentially makes it a simple and quick method to determine ICH volume, which could aid in clinical decision-making and assessing patient eligibility for clinical trials. However, the accuracy of automated ICH volume measurements with StrokeViewer has not been studied. Given the increasing use of StrokeViewer and similar AI applications, it is crucial to establish its value in determining ICH volume and compare it to other commonly used methods before it can be implemented in clinical practice.

The aim of this study is to investigate the level of agreement between fully automated haematoma volume measurement provided by StrokeViewer and the reference method (semi-automatic segmentation with Brainlab) in patients with spontaneous, supratentorial ICH. Additionally, we report the agreement between the ABC/2 method—as a widely used estimation technique—and both StrokeViewer’s output and the reference standard, to provide a comparative perspective. Furthermore, we investigate which factors might influence the agreement with the reference standard.

## Patients and methods

### Study design and patient selection

We conducted a single-centre retrospective cross-sectional study. We collected clinical and imaging data from 300 eligible consecutive patients admitted between November 2018 and June 2023. Patients presented either primarily in Erasmus University Medical Center or were referred by regional primary stroke centres. We included patients with spontaneous, supratentorial ICH confirmed by CT imaging, aged 18 years and older and with a symptom onset to CT time of less than 24 hours. We excluded patients with underlying macrovascular pathology or intracranial malignancies. The Institutional Review Board of the Erasmus MC approved the study protocol (MEC-2023-0097) and confirmed that the Dutch Medical Research Involving Human Subjects Act (WMO) was not applicable and the need for obtaining informed consent was waived.

### Sample size calculation

We assessed the sample size for the study according to Lu et al.^12^ We proposed that the volume estimation must be precise and that, therefore, a difference between 2 methods of 10% or less would be considered acceptable. In the Dutch ICH Surgery Trial (DIST) pilot study, the mean haematoma volume was 50 mL (SD: 23).^13^ Accurate volume estimation was critical to evaluate eligibility for intervention. A difference of 5 mL (SD: 2) would therefore be considered as an acceptable upper limit, as this reflects the precision required in both a clinical and research context. This assumption is in accordance with a previous study examining the accuracy of the ABC/2 method in data from 3 ICH trials.^7^ Based on this assumption, we calculated that at least 300 paired comparisons were needed to reach a power (1-beta) of more than 80%.

### Measurements

We used the semi-automatic segmentation software from Brainlab (SmartBrush, Brainlab AG, Munich, Germany) as the reference standard for ICH volume measurements, as it enables an accurate, threshold-based segmentation which can be manually adjusted. We additionally measured ICH volume by: (1) automatic segmentation with the StrokeViewer application, and (2) manual ABC/2 method using NCCT images. We used the thinnest slices available, either 1, 3 or 5 mm. The volume of intraventricular haemorrhage was not considered part of the actual ICH.

The StrokeViewer application software (Nicolab B.V., Amsterdam, The Netherlands) was used to assess the ICH volume by sending the non-contrast head CT to the Nicolab server. In this application, an AI stroke algorithm based on a deep learning convolutional neural network is used to detect haemorrhage.^10^ After analysis, the output is automatically sent to the electronic patient record. Additionally, output can also be accessed in a secured mobile phone and in web applications. Every individual segmentation was checked (N.C.) to see which parts of the haematoma were segmented by the StrokeViewer software.

The semi-automatic segmentation method to measure ICH volume was performed by 3 researchers (N.C., R.F., Y.H.) experienced with the Brainlab software, either through use in clinical practice, research or both and masked for the results of the other measurements. These measurements were checked by 2 expert users, a neurosurgeon (R.D.) and a neuroradiologist (R.G.). With Brainlab software, the user needs to give minimal manual input on 2 slices in different orthogonal planes, after which the software creates a 3D segmentation. After initial segmentation, adjustments can be made on 1 or more slice(s), which the software immediately applies to the whole segmentation.

Lastly, 5 researchers (N.C., B.S., R.W., C.C.K., S.S.), masked for the results of other measurement methods, measured ICH volume with the ABC/2 method.^5^ These researchers have worked as medical doctors within the neurology, neurosurgery or radiology department and have experience with stroke patients in the emergency room. They followed a standard operation procedure based on the article by Kothari et al.^5^ Since this is the level of experience users in clinical practice are likely to have, it represents the use of the ABC/2 method in clinical practice. In the formula, A represents the largest diameter (cm) of haemorrhage observed in the axial plane on CT, B represents the largest diameter (cm) perpendicular to A and C represents the estimated number of CT slices containing a certain percentage of the haematoma multiplied by the thickness (cm) of each slice. After multiplication (A·B·C), the outcome is divided by 2. Of the 300 scans, 1 researcher assessed 100 unique scans, while the other 4 each assessed 50 unique scans. To assess inter-observer variability, 4 researchers measured an additional 5–10 scans from a different batch, ensuring that around 10% of scans were analysed by 3 researchers. For further comparisons with the 2 other methods, the first ABC/2 measurement was used, while the second ABC/2 measurement was only used to determine inter-observer variability. Researchers that were involved with both the Brainlab and the ABC/2 measurements received a different set of scans for each of the methods.

### Data analysis

ICH volumes were assessed for all patients per method, resulting in 3 different measurements for each case. Since we performed the Brainlab and ABC/2 measurements ourselves, we expected no missing data for these methods. However, volume assessment using the StrokeViewer application may result in missing data, since the automated analysis might not always succeed. These cases were reported and examined to identify the causes of failure of the automated analysis.

Statistical analyses were conducted with R (R Core Team, version 4.3.1). ICH volume measurements and demographic characteristics were analysed with standard descriptive statistical analyses for all patients. Further analyses were performed only with patients with complete data for that analysis. Mean differences in ICH volumes between the measurement methods were determined with paired *t*-tests, and the Shapiro–Wilk test was used in addition to visual inspection of the density plots to assess the normality of data. The intraclass correlation coefficient was calculated to test inter-observer variability for ABC/2.

We used the Bland–Altman approach to assess the agreement between the 3 measurement techniques. We estimated the limits of agreement (LoA) defined as mean difference plus or minus 1.96 times the SD of differences. For limits of agreement, we posed a difference of 10% or less as clinically acceptable.

For assessment of heteroscedasticity (ie, heterogeneity of variance) we visually inspected the Bland–Altman plots and calculated the Kendall’s tau correlation coefficients. If heteroscedasticity was suspected (tau > 0.1), the volume measurements were log-transformed, and the Kendall’s tau correlation was calculated again. If tau decreased, the log-transformed LoA were back-transformed to the original scale and plotted in the conventional Bland–Altman plots as LoA as a function of the mean (*X*) for clearer interpretation.^14^ Finally, we performed subgroup analyses for patients with and without accompanying intraventricular haemorrhage (IVH), lobar or basal ganglia/thalamus ICH and volumes smaller or larger than 40 mL.

## Results

### Patient characteristics

The median age was 69 years (IQR, 57–76), 124 patients (41.3%) were women and the median NIHSS was 18 (IQR, 11–23) (Table 1). In total, 197 patients (66%) had a basal ganglia or thalamus ICH, and 148 patients (49.3%) had accompanying IVH.

**Table 1:** Baseline characteristics of 300 patients with intracerebral haemorrhage included in the study

**Characteristic**		**Missing**
Age, median (IQR)	69 (57–76)	
Sex (f), *n* (%)	124 (41%)	
Medical history of, *n* (%)		
Hypertension	132 (45%)	6 (2%)
Diabetes mellitus	47 (16%)	6 (2%)
Previous stroke^a^	75 (26%)	6 (2%)
Ischemic stroke (IS)	20 (27%)	
TIA	35 (47%)	
Previous ICH	28 (37%)	
Medication at baseline, *n* (%)		
Antihypertensive agent	119 (42%)	14 (5%)
Antiplatelet agent	87 (30%)	11 (4%)
Vitamin K antagonist	30 (10%)	10 (3%)
DOAC	24 (8%)	10 (3%)
Heparin	3 (1%)	10 (3%)
Statin	78 (27%)	12 (4%)
Pre-ICH mRS score		75 (25%)
0	109 (48%)	
1	50 (22%)	
2	32 (14%)	
3	27 (12%)	
4	6 (3%)	
5	1 (0.4%)	
GCS (on admission), median (IQR)	11 (7; 14)	2 (0.7%)
NIHSS score (on admission), median (IQR)	18 (11; 23)	67 (22%)
Hemiparesis, *n* (%)	216 (77%)	19 (6%)
Aphasia, *n* (%)	160 (62%)	43 (14%)
Hemianopia, *n* (%)	130 (53%)	54 (18%)
Baseline NCCT scan		
ICH volume^b^, mL, median (IQR)	26.0 (9.3–59.2)	
Basal ganglia or thalamus, *n* (%)	197 (66%)	
Left hemispheric location, *n* (%)	162 (54%)	
IVH present, *n* (%)	152 (51%)	
Multiple ICH, *n* (%)	7 (2%)	

^a^3 patients with ICH&IS, 2 with IS&TIA, 1 with ICH&TIA, 1 with ICH&IS&TIA.

^b^Brainlab.

Abbreviations: DOAC = direct oral anticoagulant; GCS = Glasgow Coma Scale; IVH = intraventricular haemorrhage; *n* = number; NCCT = non-contrast CT; SD = standard deviation.

### Imaging results

All CT scans performed in our cohort had a slice thickness 5 mm or less and most were 1 mm. The median ICH volumes were 40.4 mL with StrokeViewer (IQR, 21.2–74.7), 26.0 mL with Brainlab (IQR, 9.3–59.2) and 26.7 mL with ABC/2 (IQR, 10.9–68.9) (Table 2). In 28/300 cases (9.3%), the ABC/2 was performed by 2 researchers. The overall Intraclass correlation coefficient was 0.97, corresponding to an excellent inter-observer agreement.

**Table 2:** ICH volume measurements

**Measurement**	**Brainlab**	**ABC/2** ^**a**^	**StrokeViewer**
ICH volume (all patients)^b^			
Mean	39.9	50.0	55.0
Median (IQR)	26.0 (9.3–59.2)	26.7 (10.9–68.9)	40.4 (21.2;74.7)
ICH volume (for all complete cases, *n* = 250)			
Mean	43.8	55.4	55.0
Median (IQR)	29.3 (12.5–63.2)	32.2 (13.6–71.9)	40.0 (21.2;74.7)
ICH volume (in cases with missing StrokeViewer data, *n* = 50)			
Mean	20.6	23.1	–
Median (IQR)	3.5 (1.3–36.1)	4.0 (1.6–26.5)	
**Subgroup: patients without IVH**			
ICH volume (all patients, *n* = 148)			
Mean	34.2	41.3	36.8
Median (IQR)	23.9 (7.8–52.4)	26.1 (10.6–62.1)	29.6 (14.4–53.4)
ICH volume (for all complete cases, *n* = 112)			
Mean	38.9	47.4	36.8
Median (IQR)	27.9 (13.6–56.1)	31.7 (15.1–65.5)	29.6 (14.4–53.4)

^a^First measurement.

^b^ *n* = 300 for Brainlab and ABC/2 and *n* = 250 for StrokeViewer.

Abbreviations: IVH = intraventricular haemorrhage; *n* = number.

StrokeViewer detected ICH in 250 of 300 scans (83%) of confirmed supratentorial ICH. Of the 50 scans without ICH detection, 26 of the haematomas were not recognised. In the other 24, StrokeViewer notified that it was unable to perform the analysis. In the 50 patients in whom the ICH was not detected, the median ICH volume, as measured with Brainlab, was 3.5 mL (IQR, 1.3–36.1) (Table 2).

In addition to detecting the ICH, StrokeViewer also segmented other hyperdensities, such as: IVH (*n* = 131), dura (*n* = 73), bony skull base (*n* = 13), vascular structures (*n* = 9), choroid plexus calcifications (*n* = 5) and globus pallidus calcifications (*n* = 3). Additionally, 134 segmentations included unspecified small hyperdense dots within the parenchyma. In most cases, these dots were thought to be either atherosclerotic vascular structures or a result of noise caused by lower scan quality. In 55 cases, segmentation captured only a portion of the haematoma, which occurred most frequently in haematomas with varying densities. Illustrative images are shown in Figure S1.

### Assessment of heteroscedasticity

**Figure 1:**
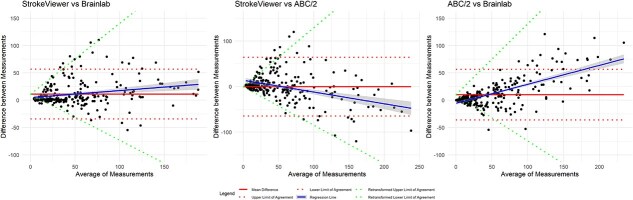
Bland–Altman plots with back transformed LoA as a function of the mean. Bland–Altman plots on the original scale for mean absolute difference (red solid line) with LoA (red dotted line), regression line (blue solid line) and back transformed LoA, where the back transformed LoA are expressed as a function of the mean (green dotted line) for (1) Agreement between StrokeViewer and Brainlab. (2) Agreement between StrokeViewer and ABC/2. (3) Agreement between ABC/2 and Brainlab. Abbreviation: LoA = limits of agreement.

**Figure 2:**
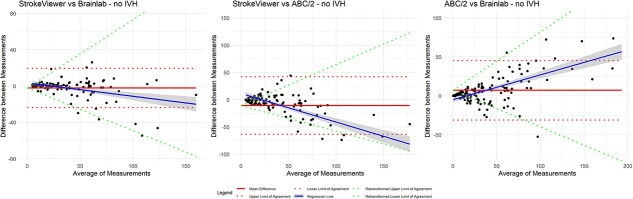
Bland–Altman plots with back transformed LoA as a function of the mean for patients without IVH. Bland–Altman plots for patients without IVH on the original scale for mean absolute difference (red solid line) with LoA (red dotted line), regression line (blue solid line) and back transformed LoA, where the back transformed LoA are expressed as a function of the mean (green dotted line) for (1) Agreement between StrokeViewer and Brainlab. (2) Agreement between StrokeViewer and ABC/2. (3) Agreement between ABC/2 and Brainlab. Abbreviations: IVH = intraventricular haemorrhage; LoA = limits of agreement.

Visual inspection of Bland–Altman plots showed heteroscedasticity for all 3 comparisons with absolute differences (Figure S2) and for the comparisons in the subgroup analysis of patients without IVH (Figure S3). This was confirmed with Kendall’s tau correlation coefficients (Tables S1 and S2). Therefore, the measurements were log-transformed (Figures S4 and S5). The final Bland–Altman plots, after back-transformation to the original scale, are shown in Figures 1 and 2.

### Agreement of ICH volume measurements

Based on the assessment of heteroscedasticity, log-transformed LoA were added to the Bland–Altman plots with the absolute differences between StrokeViewer and Brainlab, StrokeViewer and ABC/2, as well as ABC/2 and Brainlab (Figure 1). Bland–Altman plots with the corresponding percentage differences are shown in Figure S6.

Between StrokeViewer and Brainlab, the mean absolute difference was 11.2 mL (LoA, −34.3 to 56.7, with the back-transformed LoA as a function of the mean of −0.84*X* to 1.30*X*, where *X* is the mean) and the percentage difference was 27.2% (LoA, −66.7 to 121.2). Between StrokeViewer and ABC/2 the mean absolute difference was −0.42 mL (LoA, −65.0 to 64.9, with the back transformed LoA as a function of the mean of −1.10*X* to 1.31*X*) and the percentage difference was 12.4% (LoA, −101.9 to 126.7). Between ABC/2 and Brainlab the mean absolute difference was 10.1 mL (LoA, −36.2 to 56.4, with the back transformed LoA as a function of the mean of −0.52*X* to 0.79*X*) and the percentage difference was 14.4% (LoA, −51.5 to 80.3).

Subgroup analysis for ICH location (lobar vs basal ganglia/thalamus) and volume (ICH < 40 mL vs ICH > 40 mL) showed a slightly better agreement in lobar ICH or smaller ICH volume (<40 mL) between StrokeViewer and Brainlab (Figures S7 and S8).

### Effect of intraventricular haemorrhage

Subgroup analysis of patients without IVH revealed a median ICH volume of 29.6 mL with StrokeViewer (IQR, 14.4–53.4), 23.9 mL with Brainlab (IQR, 7.8–51.5) and 26.1 mL with ABC/2 (IQR, 10.6–62.1). The Bland–Altman plots, comparing StrokeViewer to Brainlab, StrokeViewer to ABC/2 and ABC/2 to Brainlab, are shown in Figure 2.

Between StrokeViewer and Brainlab, the mean absolute difference was −2.1 mL (LoA, −23.7 to 19.5, with the back transformed LoA as a function of the mean of −0.42*X* to 0.40*X*) and the percentage difference was −1.0% (LoA, −41.7 to 39.8). Between StrokeViewer and ABC/2 the mean absolute difference was −10.6 mL (LoA, −63.6 to 42.4, with back transformed LoA as a function of the mean of −0.88*X* to 0.61*X*) and the percentage difference was −15.4% (LoA, −90.9 to 60.2). Between ABC/2 and Brainlab the mean absolute difference was 7.1 mL (LoA, −31.1 to 45.3), with back transformed LoA as a function to the mean of −0.47*X* to 0.75*X*) and the percentage difference was 15.4% (LoA, −46.7 to 77.4).

## Discussion

This study demonstrated substantial disagreement between 3 methods for ICH volume measurement: StrokeViewer, Brainlab (reference standard) and ABC/2. We found a mean difference of 11.2 mL (LoA, −34.3 to 56.7) between StrokeViewer and Brainlab, −0.42 mL (LoA, −65.0 to 64.9) between StrokeViewer and ABC/2 and 10.1 mL (LoA, −36.2 to 56.4) between ABC/2 and Brainlab. When considering a clinical limit of acceptance of 10%, neither StrokeViewer nor ABC/2 was in agreement with the reference standard of semi-automatic segmentation with Brainlab. These observed differences highlight the importance of understanding variations between measurement techniques to ensure accurate and reliable ICH volume estimation.

The most substantial differences between the measurements were found in patients with IVH, as StrokeViewer included both IVH and ICH into the segmentation.

In a subgroup analysis performed with patients without IVH, there was better agreement between the methods. However, the limits of agreement remained greater than clinically acceptable. A possible explanation for the discrepancies observed when comparing StrokeViewer to the reference standard, is its inclusion of hyperdense structures, such as the dura or vascular structures. This led to an overestimation of the ICH volume. Conversely, in 55 cases, StrokeViewer segmented the ICH only partially, resulting in an underestimation of the volume.

A previous study, which validated a novel, fully automated ICH volume quantification software (qER-NCCT) in a Swedish population, reported excellent agreement in volume quantification between the fully automated software (qER-NCCT) and manual segmentation (their reference standard).^8^ However, Bland–Altman analysis showed significant differences between the methods with a mean difference of 8.3 mL and limits of agreement of −15 to 32, which exceed our proposed clinically acceptable limits of agreement. The fully automated software used in this study missed a small percentage of ICH (all small haematoma volumes [≤1 mm in diameter]), which was in concordance with our findings when analysing StrokeViewer’s performance.

Subgroup analysis of basal ganglia/thalamus ICH showed a better agreement between ABC/2 and Brainlab, which can be explained by the more ellipsoid shape of basal ganglia/thalamus ICH, which is more suited for the ABC/2 method. In these ICH, StrokeViewer overestimated the volume, probably due to the accompanying IVH, which was present in a substantial part of these patients. In patients with lobar ICH, there was a better agreement between the 3 methods, which can be partly due to few patients with lobar ICH and IVH. However, for both subgroups, the differences were still not clinically acceptable (>10% difference). Furthermore, while subgroups analysis of patients with smaller ICH (<40 mL) showed better agreement between StrokeViewer and Brainlab, these differences were also not clinically acceptable (>10% difference).

Although ABC/2 is considered an accepted and valid method for measuring ICH volume, our findings revealed poor agreement between the ABC/2 method and our reference method. The estimation of the ICH volume with ABC/2 can be carried out by any clinician within a few minutes. However, the ABC/2 method assumes that the haematoma has an ellipsoid shape, while haematomas are often more irregularly shaped. As a consequence, ABC/2 overestimates volumes in irregularly shaped haematomas, which can be challenging when volume measurement is essential for treatment decisions, or when it is used as an inclusion criterion in a time sensitive trial. When volume measurements are used as proxies of treatment effect, the method used to estimate ICH volumes should be reliable and valid.^6^^,^^7^ Despite these limitations, ABC/2 can still be used to obtain a quick first estimation of the ICH volume, as long as clinicians are aware that it is a rough estimation. Notably, we did find good inter-observer agreement of ABC/2 in accordance with previous literature.^15^

To our knowledge, this is the first study conducted to evaluate the performance of StrokeViewer application for ICH volume measurements. The baseline characteristics of our study population are similar compared to other studies investigating the accuracy of ICH volume measurement methods.^6^^,^^7^ Since we included patients that presented directly to our university medical centre as well as patients that were referred from primary stroke centres, this study represents the broader population. Patients with very small ICH (<10 cc) or only limited symptoms may be underrepresented, because they may not have been referred. Another strength of our study is that we included patients with a large range of ICH volumes.

A limitation of our study is that, despite the inclusion of a wide variety of ICH volumes, we found a skewed distribution towards lower volumes. While this is expected in the ICH population, this led to methodological challenges and reduced agreement across the spectrum, as the largest discrepancies were observed in cases with larger haematomas. However, the Bland–Altman plots also showed some disagreement between methods when evaluating the smaller haematomas.

Next, baseline NIHSS and mRS were missing for about a quarter of the patients. To account for this, we included data on hemiparesis, aphasia and hemianopia, which was available for more patients. Since we only used the NIHSS and mRS to present the baseline characteristics of the population, and not for further analysis, this did not influence our results.

Another limitation of this study was the inability to perform a complete analysis of all the scans using StrokeViewer, which may have impacted the results. In 24 scans, Strokeviewer could not perform an analysis, partly due to missing raw scan data. In an additional 26 scans, the ICH was not detected although the analysis was completed. This means that only 250 scans were available for comparison with Brainlab and ABC/2 and that our analysis provides an optimistic view of the accuracy of StrokeViewer. Most of the scans missing from StrokeViewer analysis concerned patients with small haematomas. This could be attributed to possible lower contrast or less distinct features present in small haematomas on the scans.

Furthermore, StrokeViewer was not able to distinguish IVH from ICH. Approximately 50% of our ICH patients had extension of the haematoma into the ventricular system, which resulted in inaccurate measurements, resulting in overestimation, of ICH volume in this subgroup. Moreover, the segmented area in StrokeViewer cannot be adjusted by the clinician, contrary to semi-automated segmentation in Brainlab. To improve StrokeViewer accuracy of ICH volume measurements, the model could be trained on more ICH data, in addition to ischaemic stroke data. The limitation of fully automatic segmentation regarding differentiating IVH from ICH, has also been described in other studies, which emphasises the need for development and refinement of algorithms for ICH with concurrent IVH.^9^

## Conclusion

At this moment, the results of the ICH segmentation by the StrokeViewer application cannot be directly used for clinical decision-making without additional visual verification. Currently, its segmentation accuracy is insufficient for determining patient eligibility for clinical trials based on ICH volume. However, after verification of the segmentation, StrokeViewer’s results might still aid in assessing the prognosis and determining treatment choice for patients in the acute phase.

## Supplementary Material

aakaf020_Figures_and_supplement_revised_20251013_track_changes

aakaf020_Colmer_25-0759_VA

aakaf020_Figures_and_supplement_revised_20251013_clean In the supplementary material, we provide the complete data analysis, including the assessment of heteroscedasticity, the conventional Bland–Altman plots and the log-transformed measurements and Bland–Altman plots.

## Data Availability

The research data can be made available upon request.
