# Supplementary material for: Validity of intracerebral haemorrhage volume assessment: comparison of fully automated segmentation analysis with manual ABC/2 and semi-automated measurement
Source: Eur Stroke J. 2026 Jan 1;11(1):aakaf020. doi: 10.1093/esj/aakaf020 (PMC12866654; doi:10.1093/esj/aakaf020)
Supplement: aakaf020_Figures_and_supplement_revised_20251013_track_changes [file aakaf020_figures_and_supplement_revised_20251013_track_changes.docx]

**SUPPLEMENTARY MATERIAL**

**Supplementary Table 1: Mean differences for the original measurements and the log-transformed measurements with assessment of heteroscedasticity**

|  | **N** | **Mean difference** | **95%CI**  **(p-value)** | **Shapiro-Wilk test**  **(W (p-value))** | **Tau-correlation** Absolute difference *vs* mean |
| --- | --- | --- | --- | --- | --- |
| **Original** |  |  |  |  |  |
| StrokeViewer vs Brainlab | 250 | 11.20 | 8.31 to 14.09 (<0.005) | 0.84 (<0.005) | 0.377 |
| StrokeViewer vs ABC/2 | 250 | -0.42 | -4.52 to 3.69 (0.842) | 0.91 (<0.005) | 0.409 |
| ABC/2 vs Brainlab | 300 | 10.09 | 7.41-12.78 (<0.005) | 0.81 (<0.005) | 0.689 |
| **Log-transformed** |  |  |  |  |  |
| StrokeViewer vs Brainlab | 250 | 0.32 |  |  | -0.076 |
| StrokeViewer vs ABC/2 | 250 | 0.17 |  |  | -0.154 |
| ABC/2 vs Brainlab | 300 | 0.15 |  |  | 0.045 |

**Supplementary Table 2: Mean differences for the original measurements and the log-transformed measurements with assessment of heteroscedasticity for patients without IVH**

|  | **N** | **Mean difference** | **95%CI**  **(p-value)** | **Shapiro-Wilk test**  **(W (p-value))** | **Tau-correlation** Absolute difference *vs* mean |
| --- | --- | --- | --- | --- | --- |
| **Original** |  |  |  |  |  |
| StrokeViewer vs Brainlab | 112 | -2.08 | -4.14 to 0.022 (0.048) | 0.72 (<0.005) | 0.423 |
| StrokeViewer vs ABC/2 | 112 | -10.61 | -15.67 to -5.56 (<0.005) | 0.75 (<0.005) | 0.550 |
| ABC/2 vs Brainlab | 148 | 7.07 | 3.90-10.23 (<0.005) | 0.82 (<0.005) | 0.680 |
| **Log-transformed** |  |  |  |  |  |
| StrokeViewer vs Brainlab | 112 | -0.01 |  |  | 0.001 |
| StrokeViewer vs ABC/2 | 112 | -0.16 |  |  | 0.046 |
| ABC/2 vs Brainlab | 148 | 0.16 |  |  | 0.008 |

**Supplementary Figure 1: illustrative images of StrokeViewer analysis**


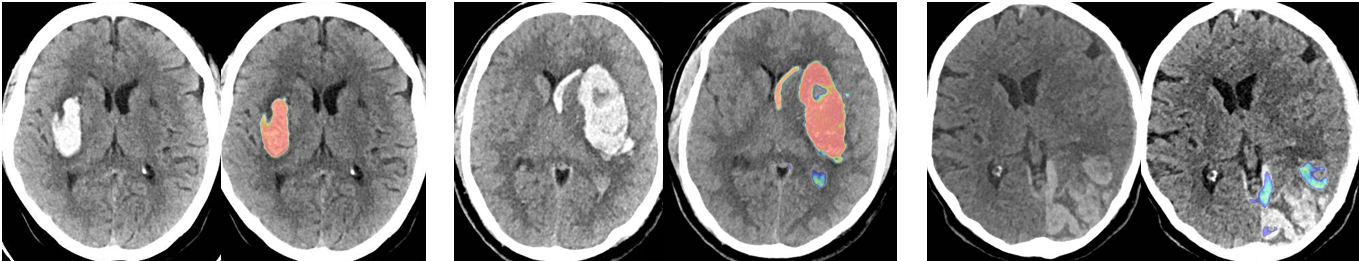


Three illustrative images of StrokeViewer analysis. Left: Good segmentation. Middle: Inclusion of IVH in the segmentation. Right: Only part of the ICH is segmented.

**Supplementary Figure 2: Conventional Bland-Altman plots**


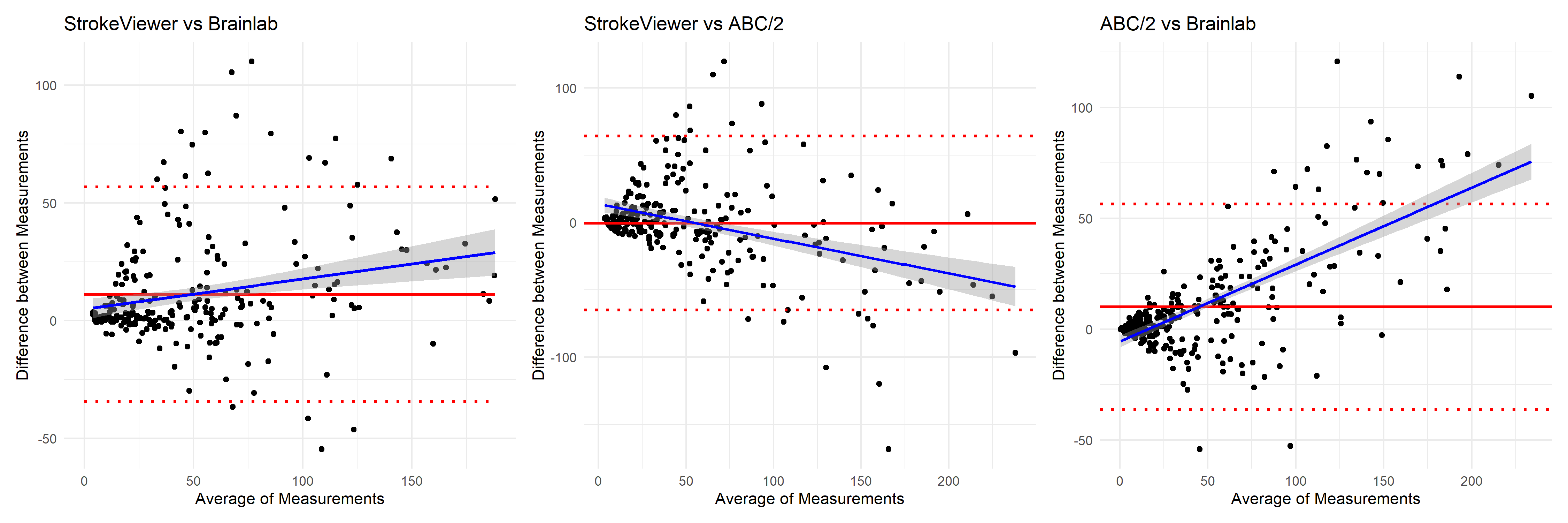


Conventional Bland-Altman plots for mean absolute difference (red solid line) with limits of agreement (LoA) (red dotted line) and regression line (blue solid line) for 1. Agreement between StrokeViewer and Brainlab. 2. Agreement between StrokeViewer and ABC/2. 3. Agreement between ABC/2 and Brainlab.

**Supplementary Figure 3: Conventional Bland-Altman plots for patients without IVH**


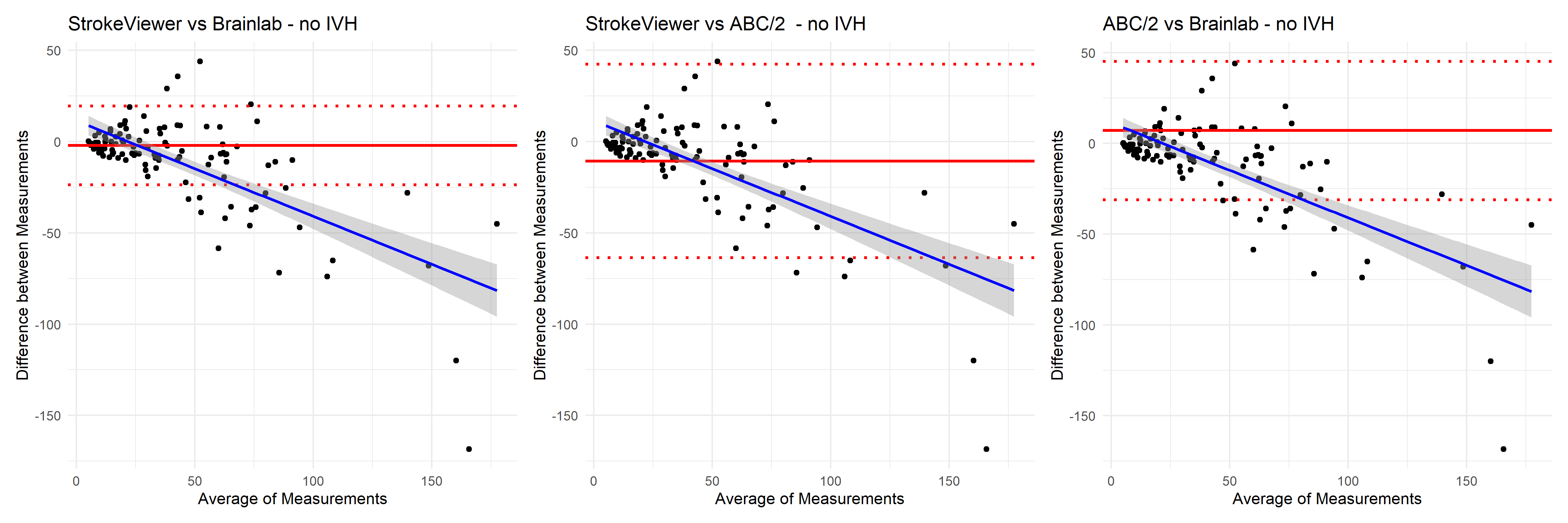


Conventional Bland-Altman plots for patients without IVH for mean absolute difference (red solid line) with limits of agreement (LoA) (red dotted line) and regression line (blue solid line) for 1. Agreement between StrokeViewer and Brainlab. 2. Agreement between StrokeViewer and ABC/2. 3. Agreement between ABC/2 and Brainlab.

**Supplementary Figure 4: Log-transformed Bland-Altman plots**


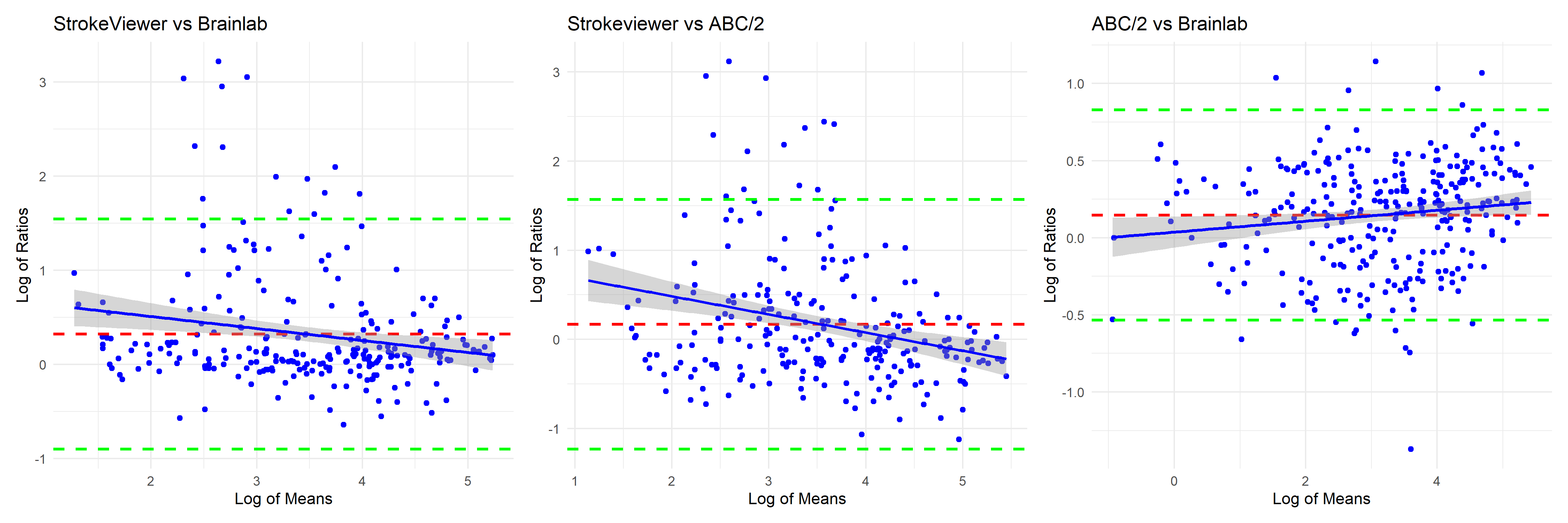


Log transformed Bland-Altman plots for mean absolute difference (red solid line) with limits of agreement (LoA) (red dotted line) and regression line (blue solid line) for 1. Agreement between StrokeViewer and Brainlab. 2. Agreement between StrokeViewer and ABC/2. 3. Agreement between ABC/2 and Brainlab.

**Supplementary Figure 5: Log-transformed Bland-Altman plots for patients without IVH**


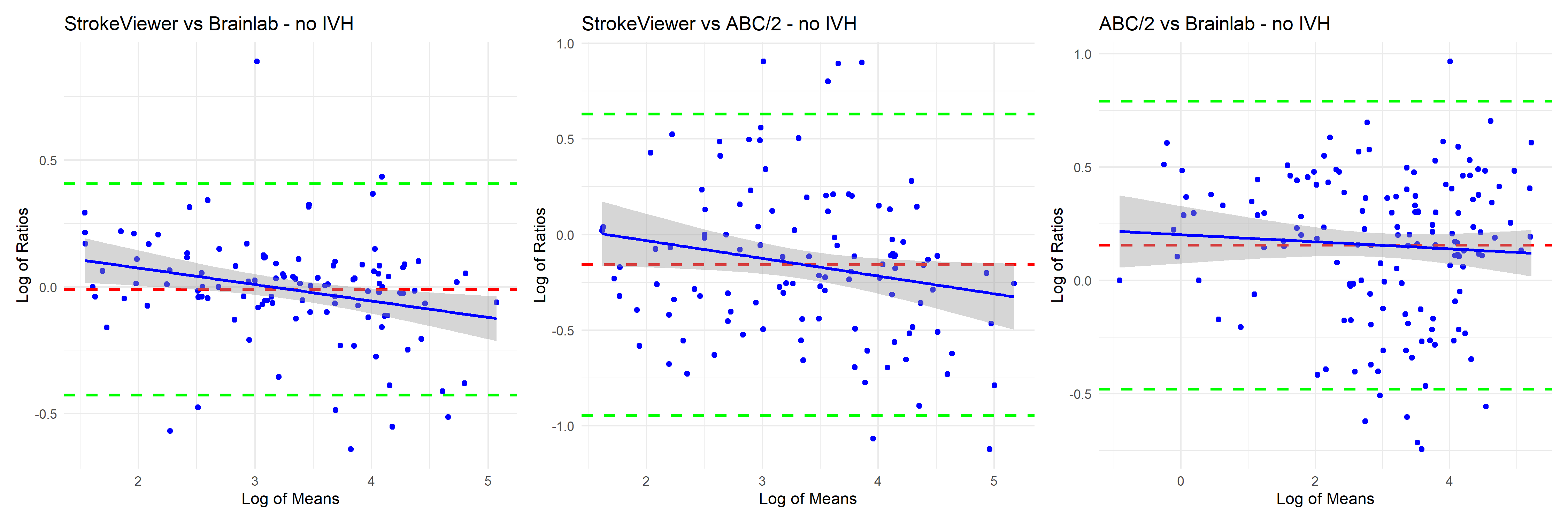


Log transformed Bland-Altman plots for patients without IVH for mean absolute difference (red solid line) with limits of agreement (LoA) (red dotted line) and regression line (blue solid line) for 1. Agreement between StrokeViewer and Brainlab. 2. Agreement between StrokeViewer and ABC/2. 3. Agreement between ABC/2 and Brainlab.

**Supplementary Figure 6: Bland-Altman plot with percentage difference**


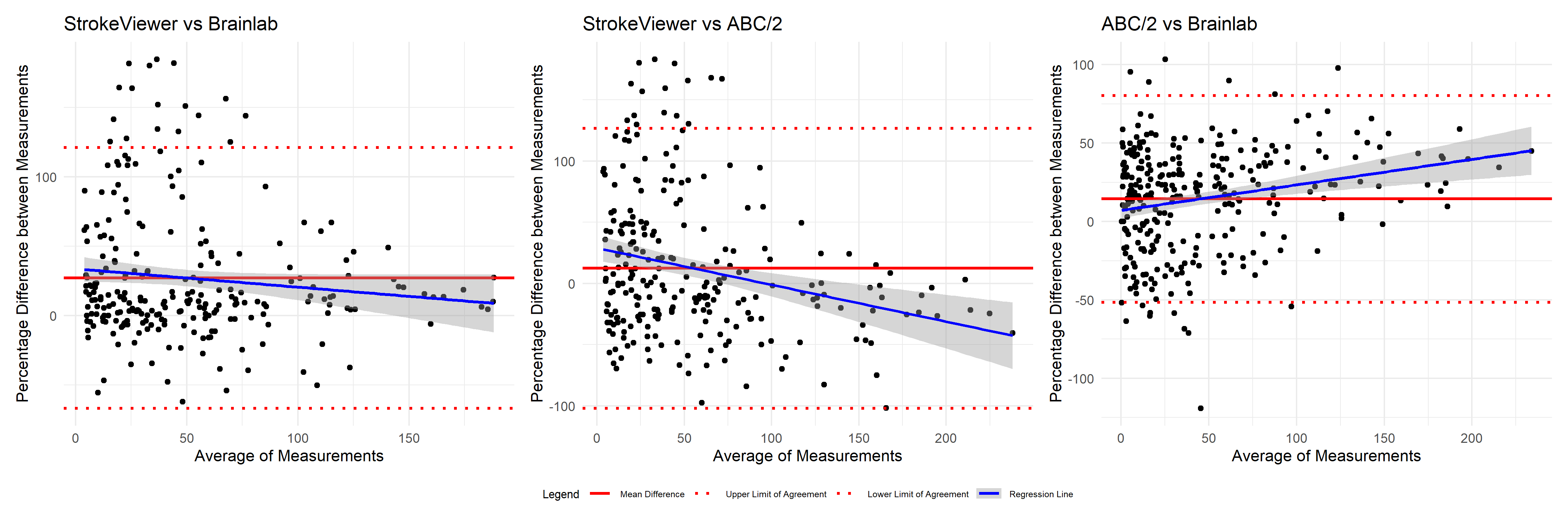


Conventional Bland-Altman plots for mean percentage difference (red solid line) with limits of agreement (LoA) (red dotted line) and regression line (blue solid line) for 1. Agreement between StrokeViewer and Brainlab. 2. Agreement between StrokeViewer and ABC/2. 3. Agreement between ABC/2 and Brainlab.

**Supplementary Figure 7: Bland-Altman plot for lobar vs basal ganglia/thalamus ICH**

**
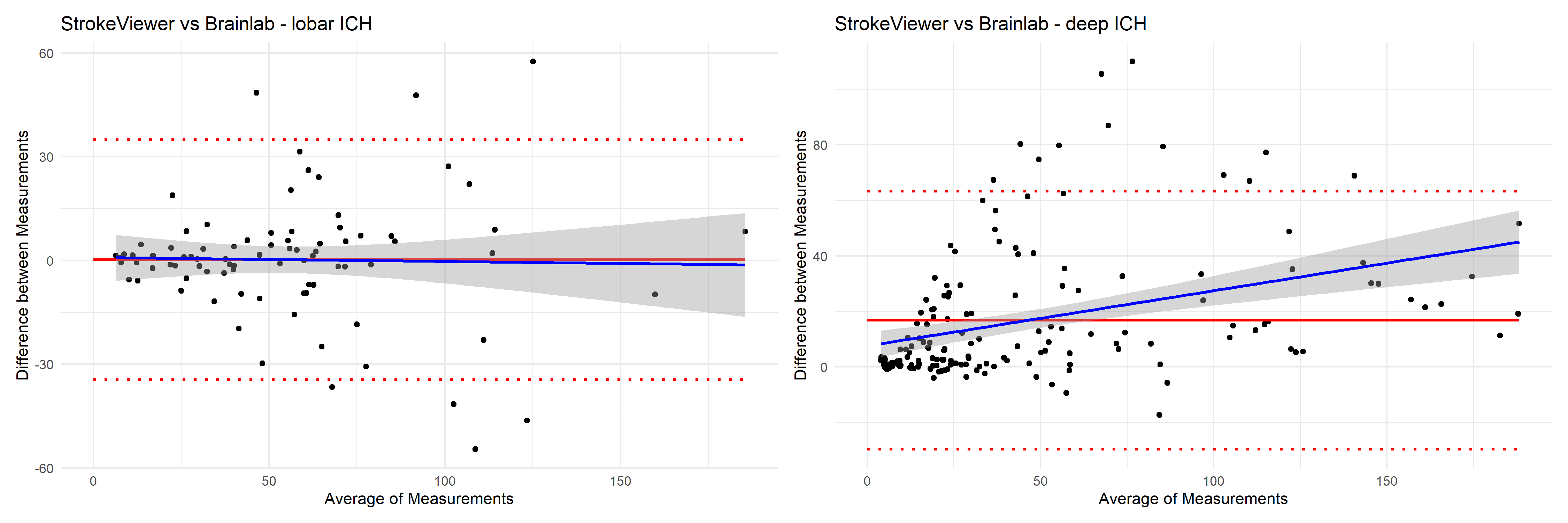
**

Conventional Bland-Altman plots for patients with lobar vs basal ganglia/thalamus ICH for mean absolute difference (red solid line) with limits of agreement (LoA) (red dotted line) and regression line (blue solid line) for agreement between StrokeViewer and Brainlab.

**Supplementary Figure 8: Bland-Altman plot for <40mL vs >40mL ICH volume**

**
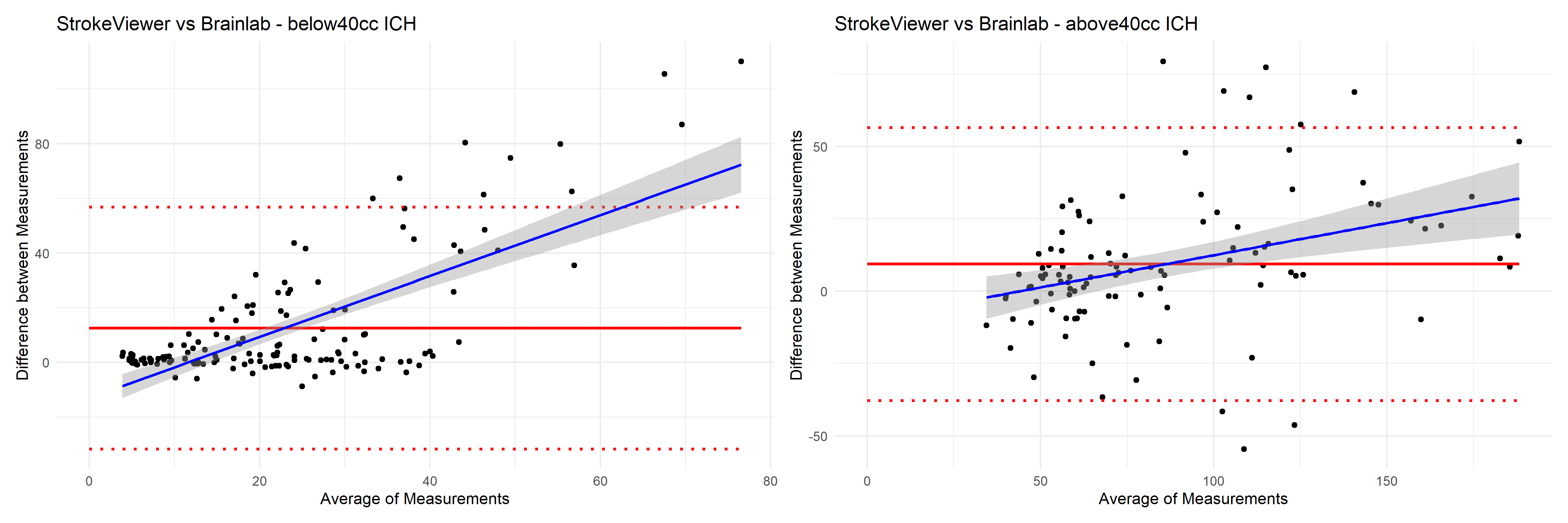
**

Conventional Bland-Altman plots for patients with ICH volume <40mL vs >40mL for mean absolute difference (red solid line) with limits of agreement (LoA) (red dotted line) and regression line (blue solid line) for agreement between StrokeViewer and Brainlab.
