# Supplementary material for: Validity of intracerebral haemorrhage volume assessment: comparison of fully automated segmentation analysis with manual ABC/2 and semi-automated measurement
Source: Eur Stroke J. 2026 Jan 1;11(1):aakaf020. doi: 10.1093/esj/aakaf020 (PMC12866654; doi:10.1093/esj/aakaf020)
Supplement: aakaf020_Colmer_25-0759_VA [file aakaf020_colmer_25-0759_va.pdf]

EUROPEAN STROKE JOURNAL

Validity of intracerebral haemorrhage volume assessment: comparison of fully automated segmentation analysis with manual ABC/2 and semi-automated measurement

To assess the agreement between automated intracerebral haemorrhage (ICH) volume measurement, the ABC/2-method, and semi-automatic segmentation in patients with spontaneous supratentorial ICH

Methods

Single-centre retrospective, cross-sectional study

CT scan in patients with ICH
n = 300

Volume measurements with:
- Fully-automated StrokeViewer software
- Manual ABC/2 method
- Semi-automated Brainlab software (reference standard)

Bland-Altman analysis

Results

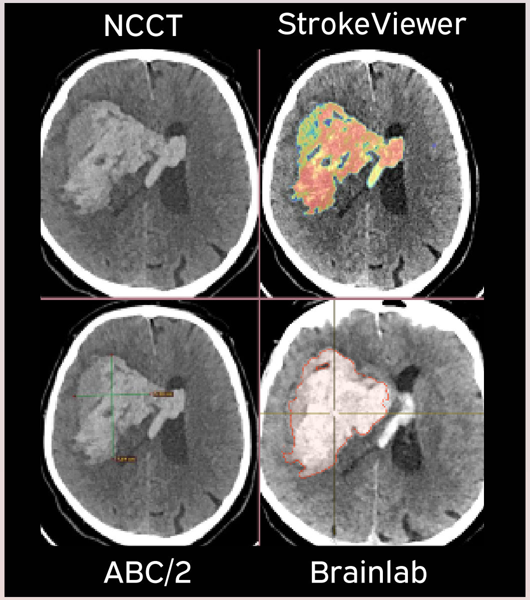

Conclusion

Neither StrokeViewer nor ABC/2 achieved clinically acceptable agreement (≤ 10%).

StrokeViewer should not be used for volume-based decisions without visual segmentation check.

Colmer, N. H. C., et al. European Stroke Journal, 2026 n.colmer@erasmusmc.nl doi.org/xx.xxxx/xxxxxxxxxxxxxx
