# Supplementary material for: Validity of intracerebral haemorrhage volume assessment: comparison of fully automated segmentation analysis with manual ABC/2 and semi-automated measurement
Source: Eur Stroke J. 2026 Jan 1;11(1):aakaf020. doi: 10.1093/esj/aakaf020 (PMC12866654; doi:10.1093/esj/aakaf020)
Supplement: aakaf020_Figures_and_supplement_revised_20251013_clean — In the supplementary material, we provide the complete data analysis, including the assessment of heteroscedasticity, the conventional Bland–Altman plots and the log-transformed measurements and Bland–Altman plots. [file aakaf020_figures_and_supplement_revised_20251013_clean.pdf]

SUPPLEMENTARY MATERIAL

Supplementary Table 1: Mean differences for the original measurements and the log-transformed measurements with assessment of heteroscedasticity

	N	Mean difference	95%CI (p-value)	Shapiro-Wilk test (W (p-value))	Tau-correlation Absolute difference *vs* mean
**Original**					
StrokeViewer vs Brainlab	250	11.20	8.31 to 14.09 (<0.005)	0.84 (<0.005)	0.377
StrokeViewer vs ABC/2	250	-0.42	-4.52 to 3.69 (0.842)	0.91 (<0.005)	0.409
ABC/2 vs Brainlab	300	10.09	7.41-12.78 (<0.005)	0.81 (<0.005)	0.689
**Log-transformed**					
StrokeViewer vs Brainlab	250	0.32			-0.076
StrokeViewer vs ABC/2	250	0.17			-0.154
ABC/2 vs Brainlab	300	0.15			0.045

Supplementary Table 2: Mean differences for the original measurements and the log-transformed measurements with assessment of heteroscedasticity for patients without IVH

	N	Mean difference	95%CI (p-value)	Shapiro-Wilk test (W (p-value))	Tau-correlation Absolute difference *vs* mean
**Original**					
StrokeViewer vs Brainlab	112	-2.08	-4.14 to 0.022 (0.048)	0.72 (<0.005)	0.423
StrokeViewer vs ABC/2	112	-10.61	-15.67 to -5.56 (<0.005)	0.75 (<0.005)	0.550
ABC/2 vs Brainlab	148	7.07	3.90-10.23 (<0.005)	0.82 (<0.005)	0.680
**Log-transformed**					
StrokeViewer vs Brainlab	112	-0.01			0.001
StrokeViewer vs ABC/2	112	-0.16			0.046
ABC/2 vs Brainlab	148	0.16			0.008

Supplementary Figure 1: illustrative images of StrokeViewer analysis

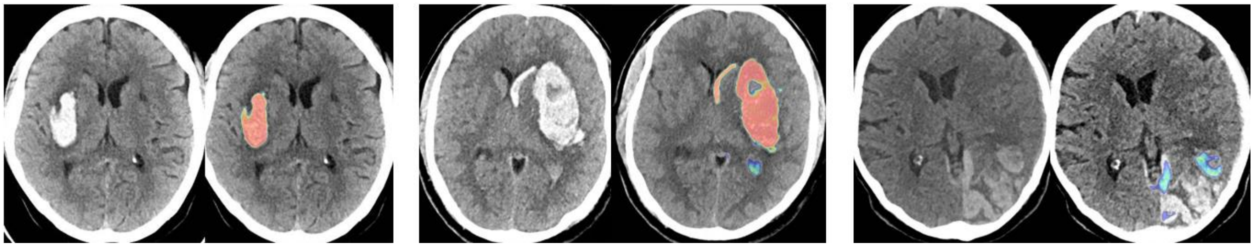

Three illustrative images of StrokeViewer analysis. Left: Good segmentation. Middle: Inclusion of IVH in the segmentation. Right: Only part of the ICH is segmented.

Supplementary Figure 2: Conventional Bland-Altman plots

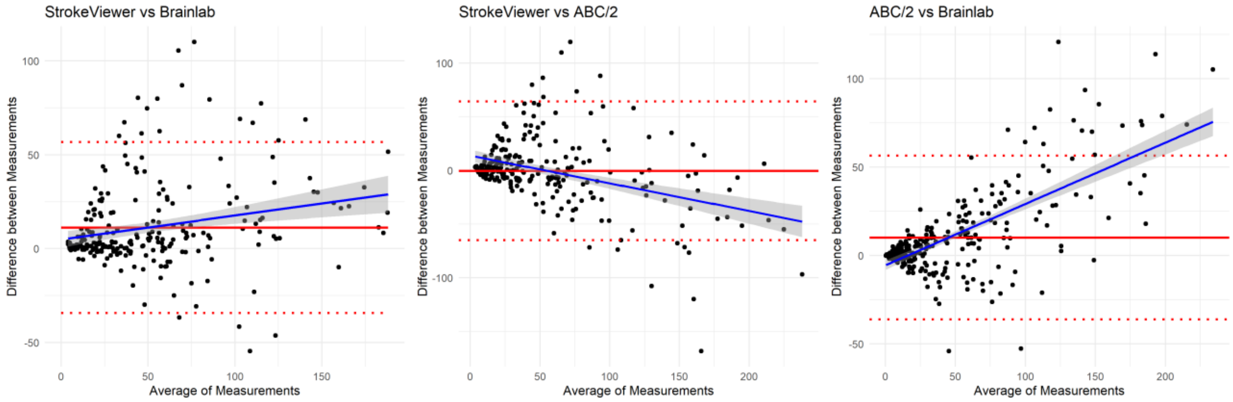

Conventional Bland-Altman plots for mean absolute difference (red solid line) with limits of agreement (LoA) (red dotted line) and regression line (blue solid line) for 1. Agreement between StrokeViewer and Brainlab. 2. Agreement between StrokeViewer and ABC/2. 3. Agreement between ABC/2 and Brainlab.

Supplementary Figure 3: Conventional Bland-Altman plots for patients without IVH

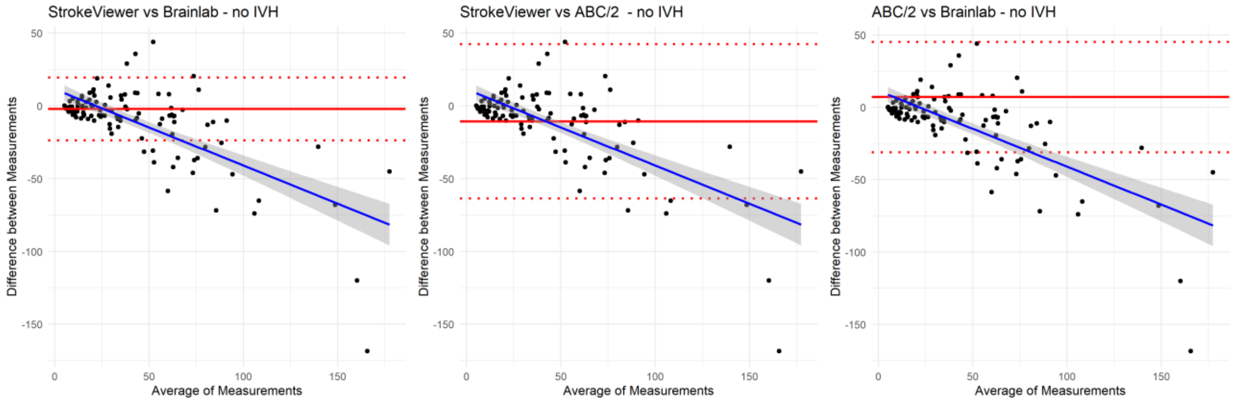

Conventional Bland-Altman plots for patients without IVH for mean absolute difference (red solid line) with limits of agreement (LoA) (red dotted line) and regression line (blue solid line) for 1. Agreement between StrokeViewer and Brainlab. 2. Agreement between StrokeViewer and ABC/2. 3. Agreement between ABC/2 and Brainlab.

Supplementary Figure 4: Log-transformed Bland-Altman plots

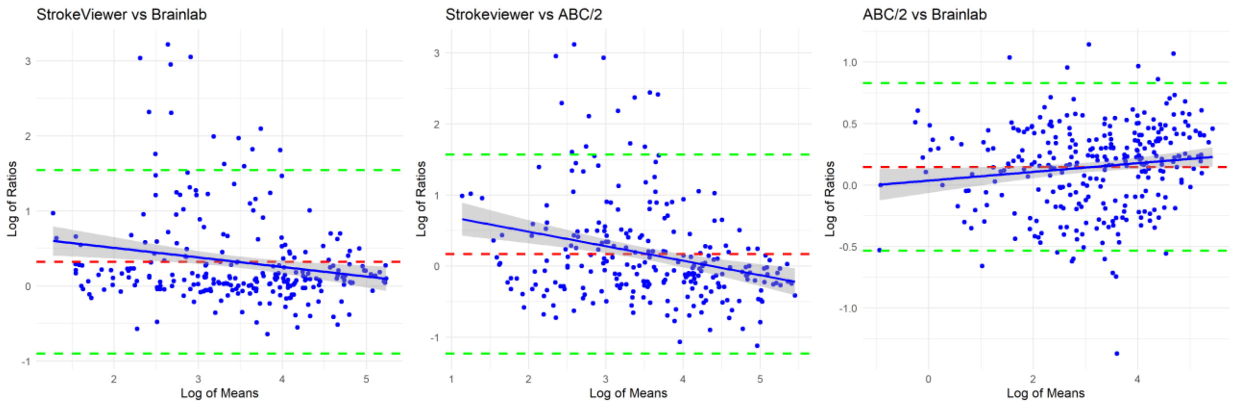

Log transformed Bland-Altman plots for mean absolute difference (red solid line) with limits of agreement (LoA) (red dotted line) and regression line (blue solid line) for 1. Agreement between StrokeViewer and Brainlab. 2. Agreement between StrokeViewer and ABC/2. 3. Agreement between ABC/2 and Brainlab.

Supplementary Figure 5: Log-transformed Bland-Altman plots for patients without IVH

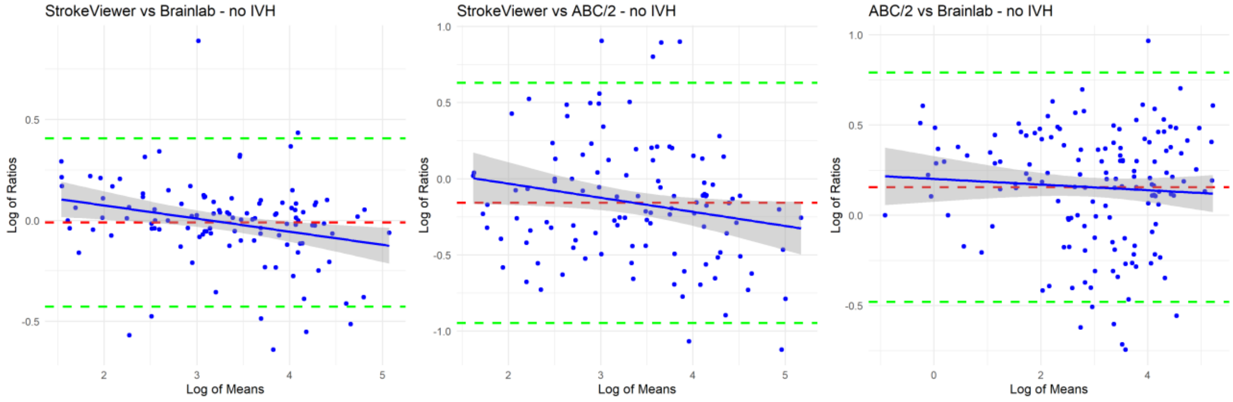

Log transformed Bland-Altman plots for patients without IVH for mean absolute difference (red solid line) with limits of agreement (LoA) (red dotted line) and regression line (blue solid line) for 1. Agreement between StrokeViewer and Brainlab. 2. Agreement between StrokeViewer and ABC/2. 3. Agreement between ABC/2 and Brainlab.

Supplementary Figure 6: Bland-Altman plot with percentage difference

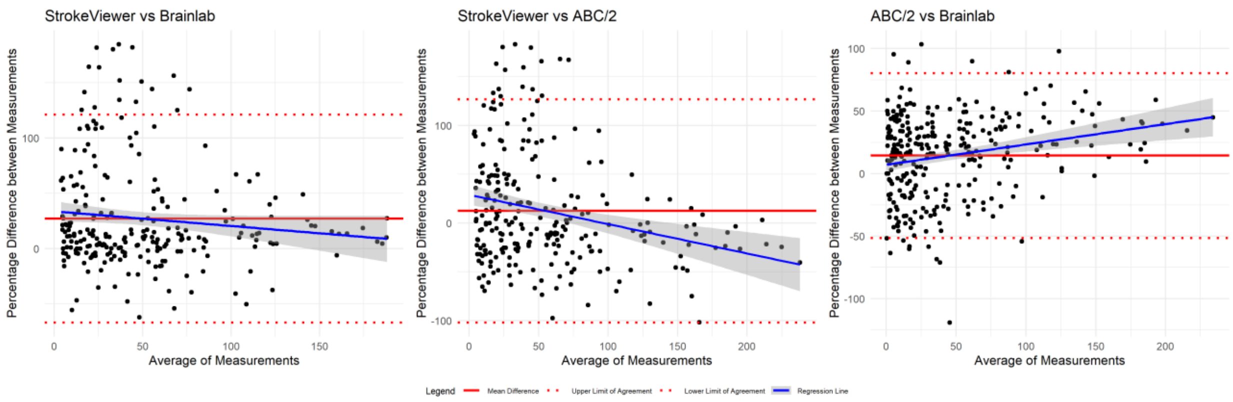

Conventional Bland-Altman plots for mean percentage difference (red solid line) with limits of agreement (LoA) (red dotted line) and regression line (blue solid line) for 1. Agreement between StrokeViewer and Brainlab. 2. Agreement between StrokeViewer and ABC/2. 3. Agreement between ABC/2 and Brainlab.

Supplementary Figure 7: Bland-Altman plot for lobar vs basal ganglia/thalamus ICH

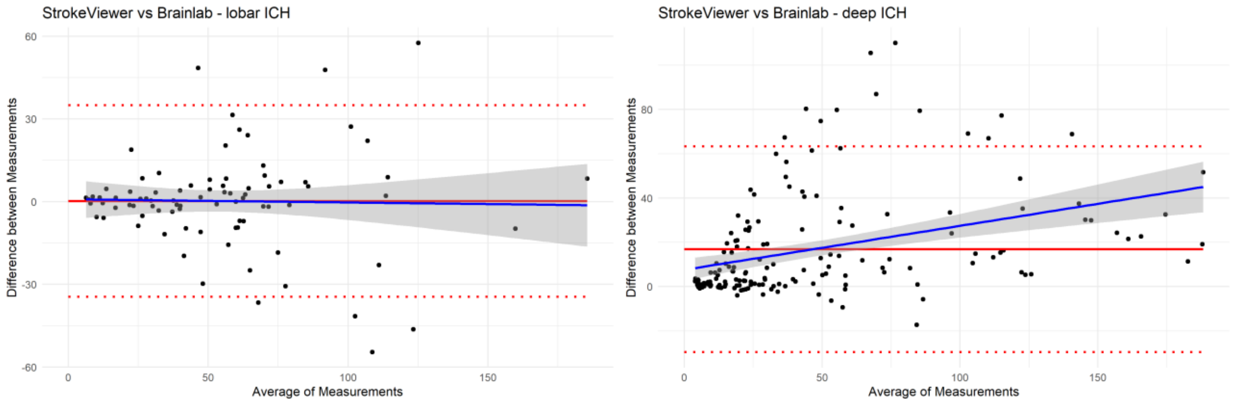

Conventional Bland-Altman plots for patients with lobar vs basal ganglia/thalamus ICH for mean absolute difference (red solid line) with limits of agreement (LoA) (red dotted line) and regression line (blue solid line) for agreement between StrokeViewer and Brainlab.

Supplementary Figure 8: Bland-Altman plot for <40mL vs >40mL ICH volume

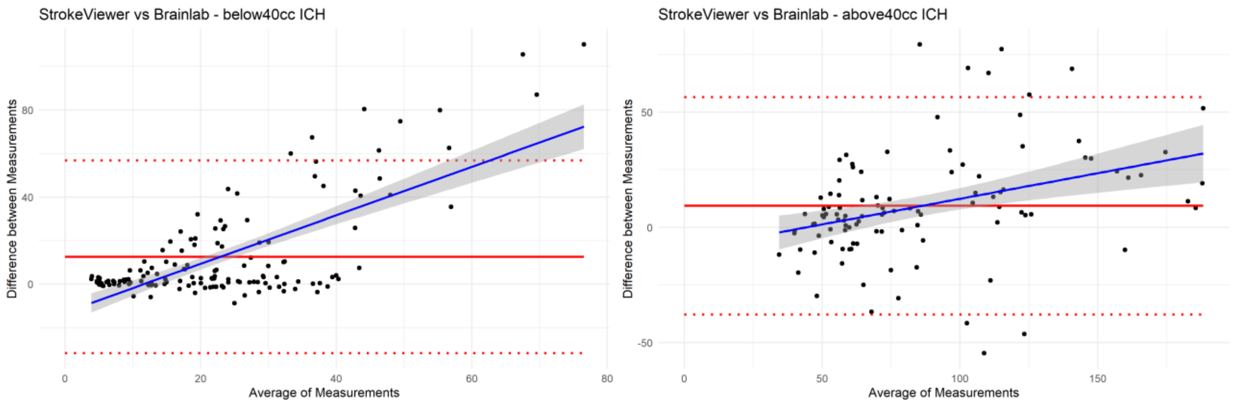

Conventional Bland-Altman plots for patients with ICH volume <40mL vs >40mL for mean absolute difference (red solid line) with limits of agreement (LoA) (red dotted line) and regression line (blue solid line) for agreement between StrokeViewer and Brainlab.
